# Effect of 15 days −6° head-down bed rest on microbial communities of supragingival plaque in young men

**DOI:** 10.3389/fmicb.2024.1331023

**Published:** 2024-01-24

**Authors:** Di Zhu, Pengyan Qiao, Qian Zhou, Hui Sun, Bingmu Xin, Bin Wu, Chuhua Tang

**Affiliations:** ^1^306th Clinical College of PLA, The Fifth Clinical College, Anhui Medical University, Beijing, China; ^2^Department of Stomatology, PLA Strategic Support Force Medical Center, Beijing, China; ^3^Engineering Research Center of Human Circadian Rhythm and Sleep, Space Science and Technology Institute, Shenzhen, China; ^4^China Astronaut Research and Training Center, Beijing, China

**Keywords:** head-down bed rest, microgravity, supragingival plaque, microbial community, 16S rRNA high-throughput sequencing

## Abstract

**Introduction:**

The microgravity environment astronauts experience during spaceflight can lead to an increased risk of oral diseases and possible changes in oral microecology. In this study, we aimed to assess changes in the microbial community of supragingival plaques to explore the effects of spaceflight microgravity environment on oral microecology.

**Methods:**

Sixteen healthy male volunteers were recruited, and supragingival plaque samples were collected under −6° head-down bed rest (HDBR) at five-time points: day 1 before HDBR; days 5, 10, and 15 of HDBR; and day 6 of recovery. Bacterial genomic DNA was sequenced using gene sequencing technology with 16S ribosomal ribonucleic acid V3–V4 hypervariable region amplification and the obtained data were analyzed bioinformatically.

**Results:**

Alpha diversity analysis showed a significant increase in species richness in supragingival plaque samples on day 15 of HDBR compared with that at pre-HDBR. Beta diversity analysis revealed that the community composition differed among the groups. Species distribution showed that, compared with those at pre-HDBR, the relative abundances of *Corynebacterium* and *Aggregatibacter* increased significantly during HDBR, while those of *Veillonella*, *Streptococcus*, and *Lautropia* decreased significantly. Moreover, compared with those at pre-HDBR, the relative abundance of *Leptotrichia* increased significantly on day 6 of recovery, whereas the relative abundances of *Porphyromonas* and *Streptococcus* decreased significantly. Network analysis showed that the interaction relationship between the dominant genera became simpler during HDBR, and the positive and negative correlations between them showed dynamic changes. Phylogenetic investigation of communities by reconstruction of unobserved states analysis showed that the amino acid metabolism function of plaque microorganisms was more enriched during HDBR.

**Discussion:**

In summary, in a 15-day simulated microgravity environment, the diversity, species distribution, interaction relationship, and metabolic function of the supragingival plaque microbial community changed, which suggests that microgravity may affect the oral microecosystem by changing the balance of supragingival plaque microbial communities and further leading to the occurrence and development of oral diseases.

## 1 Introduction

With the continuous exploration of space fields by humans, an increasing number of manned space missions have been successfully performed, and the construction of commercial spaceports is ongoing (Grenon et al., 2012). However, during space flights, the microgravity environment experienced by astronauts can affect human health and safety, including the musculoskeletal, hematopoietic, endocrine, and digestive systems (White and Averner, 2001; Graebe et al., 2004; Grimm et al., 2016).

Oral health is closely related to general health. The oral cavity is the gateway for pathogens to invade the human body and is a reservoir for infection-associated microorganisms that play a role in the spread of disease (Johnston and Dietlein, 1977). Oral dysbiosis can promote the development of oral diseases such as periodontal disease and caries, and may also affect systemic health by migration and colonization of pathogens, bacteremia, stimulating the body‘s inflammatory response and autoimmunity (Atarashi et al., 2017; Aarabi et al., 2018; Kwun et al., 2020). Therefore, oral health maintenance and monitoring of microbial communities are important for astronauts. According to a report, pain caused by pulpitis or gingivitis in astronauts during air flight may seriously affect their work efficiency (Brown et al., 1977). Astronauts experience increased dental calculus and gingival inflammation during space flights (Brown et al., 1976). In 1978, a Russian astronaut developed toothache symptoms during the last 2 weeks of a 96-day flight on Salyut 6; however, no emergency plans were made (Ball and Evans, 2001). A case of dental caries was reported in a medical incident at the Space Station MIR, which was treated with temporary fillings (Gontcharov et al., 2005). Rai (2009) confirmed through an 8-h−6° head-down-tilt bed rest experiment in 20 volunteers that a microgravity environment can cause toothache symptoms in healthy volunteers during tooth occlusion. Meanwhile, the results of space flight and simulation experiments showed that microgravity conditions could change the growth, morphology, virulence, antibiotic resistance, and secondary metabolism of microorganisms (Klaus and Howard, 2006; Wilson et al., 2007; Huang et al., 2018); change cellular interactions with the host (Foster et al., 2013); and cause negative effects on the immune system (Crucian et al., 2015). During life on the space station, astronauts’ normal microflora composition changes significantly, which results in their bodies becoming less resistant to the source of infection (Ilyin, 2005).

Microbial factors are key factors leading to oral infectious diseases such as periodontal disease and caries. Plaque biofilms are thought to initiate the development and progression of periodontal disease (Marsh, 2012). Plaque accumulation at the gingival margin may cause chronic inflammation in periodontal tissues. Disruption of the homeostatic relationship between the plaque bacteria and the host can initiate and promote periodontal disease progression (Zhang et al., 2020). A close relationship exists between the stimulatory activity of Toll-like receptors (TLR)2 and TLR4 in supragingival plaques and the periodontal status (Yoshioka et al., 2008). By counting the number of plaque microorganism clones over 2 years in 24 participants, it was found that the stability of plaque microbial composition may be a good predictor of periodontal health (Kumar et al., 2006). Furthermore, plaque microorganisms mediate the development of caries. Bacterial microorganisms in the community produce acids to erode hard dental tissues, resulting in their demineralization and, ultimately, the formation of cavities. The accumulation of supragingival plaque biofilms on teeth is usually the first manifestation of caries (Bowen, 2016). Generally, a dynamic balance exists between plaque microbes and the host, and changes in health status alter the composition of substances released by the bacteria and correspondingly alter host responses (Darveau et al., 1997).

In a previous study, our group found that the structure of salivary microbial communities in young men in the 15-day head-down bedridden state changed significantly, and differences were observed in species distribution (Sun et al., 2022). However, microbial structures can vary across different microecological sites within the oral cavity (Yang et al., 2021). Compared to saliva, plaque microbiota accumulates on non-detached teeth surfaces, which provides them with more time to develop into uniform and complex communities (Shi et al., 2018). Simultaneously, teeth have non-shedding surfaces suitable for microbial growth, which are key ecological determinants contributing to the persistence of supragingival plaque (Marsh, 2003). Various microorganisms in the plaque community interact to form complex biological networks that provide ideal sites for microbial growth and reproduction. At present, the research on the microbial community of human oral cavity in microgravity environment mainly focuses on saliva samples, while the research on supragingival plaque is very limited. Therefore, studying the microbial community structure of supragingival plaque is crucial.

The −6° head-down bed rest model is considered a well-established model for simulating the physiological effects of microgravity in astronaut spaceflight (Pavy-Le Traon et al., 2007; Hargens and Vico, 2016). In 2015, the International Academy of Astronautics (IAA) has organized a team to study and publish a standardized guideline of bed-rest studies in the spaceflight context (Sundblad et al., 2016), which clearly proposes the use of −6° head-tilt to simulate microgravity. This experiment was performed under the guidance of this guideline.

Early scientists used culture-based methods to collect supragingival plaque samples from astronauts before and after space flights and found that their microbial counts were altered (Brown et al., 1974, 1976; Johnston and Dietlein, 1977). However, due to the limitation of experimental techniques, the previously investigated microbial species are limited to a few culturable species. Meanwhile, their findings were limited to changes in the counts of specific microorganisms. These do not reflect the overall changes in the microbial community of astronauts’ supragingival plaque in the spaceflight microgravity environment, and do not report the co-occurring relationship between oral flora and predictions of microbial metabolic function. High-throughput sequencing technologies can provide new means to comprehensively understand the overall changes in the microbial community of supragingival plaques (Jagathrakshakan et al., 2015; Al-Hebshi et al., 2019). In addition, previous studies on microgravity have been limited to surveys before and after the flight or before and after simulation experiments and have not explored changes in plaque microflora at multiple time points during flight or experiments. Here, we used the −6° head-down bed rest method to simulate the microgravity environment and used 16S ribosomal ribonucleic acid (rRNA) gene sequencing technology based on the Illumina MiSeq platform to assess the changes in the microbial community of supragingival plaques at multiple time points and provide a basis for achieving precise microbiological prevention and treatment of space oral diseases in the future.

## 2 Materials and methods

### 2.1 Participants

The participants were openly recruited from the public for this study. The inclusion criteria were as follows: male; age 18–45 years; height 160–175 cm; weight 50–80 kg; physical health status. The exclusion criteria included dental caries, periodontal disease, other oral infectious diseases, smoking history, drug dependence history, mental and psychological disease history, and systemic disease history. After a physical examination and screening, 16 male participants were recruited, and none withdrew from the experiment. This study protocol conformed to the ethical principles of the Declaration of Helsinki and was approved by the Ethics Review Committee of the Space Science and Technology Institute (Shenzhen) (SISCJK202009001). All participants provided informed consent, signed an informed consent form, and received remuneration after the end of the experiment.

### 2.2 −6° head-down bed rest test

In this experiment, we used the −6° head-down bed rest (HDBR) method to simulate a microgravity environment in space. During the experiment, participants held the longitudinal axis of their body with a horizontal line at −6° head down. The experiment lasted for 28 days, including 7 days of adaptation pre-HDBR, 15 days of HDBR, and 6 days of recovery post-HDBR. Two participants were housed in the same room with beds separated by a movable curtain. Room temperature was maintained between 22°C and 26°C. The participants were provided oral hygiene education and training on the bass brushing method before the experiment. During HDBR, it was required that: (1) participants perform daily activities in bed, such as eating, bathing, and urinating; they could turn on their sides but not get up. (2) The participants woke up at 07:00 and retired at 22:30 every day. The schedule of the daily activities was strictly controlled. (3) Participants were provided a uniform diet according to nutritional standards by dietitians. No beverage, except pure water, was offered. (4) Specialists monitored the participants’ physical conditions and physiological changes to ensure that their physical indicators were within normal limits. This experiment was grouped according to 5-time points: day 1 before HDBR (AP group), during HDBR: day 5 of HDBR (BP group), day 10 of HDBR (CP group), day 15 of HDBR (DP group), and day 6 of recovery (EP group).

### 2.3 Samples collection

Supragingival plaque samples were collected at 9 a.m. Participants were asked to rinse their mouths and remove food debris. Following moisture removal using cotton rolls, supragingival plaque samples were collected from the buccal, lingual, and interproximal surfaces of six teeth, including the maxillary and mandibular first molars, the right maxillary central incisor, and the left mandibular central incisor, using sterile dental probes. The samples were placed in 1.5 mL Eppendorf tubes containing 1 mL of pre-chilled phosphate buffered saline, transferred on an ice bath, and stored in a−80°C freezer.

### 2.4 DNA extraction and purification

Target deoxyribonucleic acid (DNA) sequences in the samples were obtained using the cetyltrimethylammonium bromide (CTAB) method, and the purity and concentration of the DNA were detected using 1% agarose gel electrophoresis. An appropriate amount of DNA and a certain amount of sterile water were mixed in a centrifuge tube to dilute the sample to 1 ng/μl. Diluted genomic DNA was used as a template for PCR amplification using forward primer 341F and reverse primer 806R for the 16S rRNA V3–V4 region. PCR reactions used 15 μL Phusion® High-Fidelity PCR Master Mix (New England Biolabs, Ipswich, MA, USA), 2 μL forward and reverse primers and approximately 10 ng of template DNA. Thermal cycling consisted of initial denaturation at 98°C for 1 min, followed by 30 cycles of denaturation at 98°C for 10 s, annealing at 50°C for 30 s, and elongation at 72°C for 30 s. Finally, denaturation was performed at 72°C for 5 min. Samples were mixed equally with different tags and mixed thoroughly, and PCR amplification products were detected by 2% agarose gel electrophoresis. Target bands were tapped to obtain the recovered products. The products recovered by tapping were purified using the GeneJET Gel Extraction Kit (Qiagen, Hilden, Germany).

### 2.5 Sequencing and data analysis

Library construction was performed using a TruSeq® DNA polymerase chain reaction (PCR)-free sample preparation kit (Illumina, CA, USA). A Qubit fluorometer (Thermo Fisher, Carlsbad, CA, USA) and quantitative-PCR (Q-PCR) analysis were used to evaluate library quality. After the library was qualified, it was processed for sequencing using the Illumina NovaSeq platform (Nevogene, Beijing, China). Each data sample was split from the offboard data according to the barcode and PCR amplification primer sequences, and the sequencing reads of each sample were spliced, controlled, and filtered using FLASH (v1.2.7) after truncating the barcode and primer sequences to obtain clean tags (Magoč and Salzberg, 2011; Bokulich et al., 2013). The quality control process was completed according to the method described by Qiime (v1.9.1) (Caporaso et al., 2010; Poncheewin et al., 2019), and effective tags were obtained (Rognes et al., 2016). All effective tags were clustered using Uparse software (v7.0.1001) to cluster sequences into operational taxonomic units (OTUs) based on the similarity between sequences with 97% consistency (Haas et al., 2011). With SILVA based on the Mothur algorithm, the database performs species annotation analysis of OTUs sequences (setting a threshold of 0.8–1) to obtain the microbial community composition of each sample at different taxonomic levels (Edgar, 2013; Quast et al., 2013).^1^ Species accumulation curves were plotted using the R software (v2.15.3). Observed species, Chao1, ACE, Shannon, Simpson, and good coverage indices were calculated using the Qiime software based on species annotation. Fast multiple sequence alignment was performed using the MUSCLE software (v3.8.31) to obtain phylogenic relationships for representative sequences of all OTUs (Edgar, 2004). Principal coordinate analysis (PCoA) was performed by calculating the UniFrac distances using Qiime software, using phylogenetic relationships among OTUs (Lozupone et al., 2011). An unweighted pair-group method with an arithmetic mean (UPGMA) sample cluster tree was constructed. Multidimensional data were visualized by performing non-metric multidimensional scaling (NMDS) analysis based on the Bray-Curtis distance (Lozupone et al., 2007). The corresponding images were drawn using R software. Analysis of dissimilarities (ADONIS), analysis of similarities (ANOSIM), and analysis of molecular variance (AMOVA) were used to test the significance of the differences in community structure among the groups (Roewer et al., 1996; Chapman and Underwood, 1999; Anderson, 2001). Statistical analyses were performed using the SPSS software (v21.0.0.0). To determine whether species diversity and relative abundance differed among groups, we performed normality tests using the Shapiro–Wilk method, one-way analysis of variance (ANOVA), and Tukey multiple tests if a normal distribution was met; otherwise, the Friedman rank sum test and Dunn’s multiple tests were used. GraphPad Prism (v8.0.1) and R software were used for mapping. Network relationships among the genera were plotted using Spearman’s correlation coefficient and Graphviz software (v2.38.0). Significantly different metabolic pathways were identified by the phylogenetic investigation of communities by reconstruction of unobserved states (PICRUSt). Differences in metabolic function between groups were analyzed using a paired sample t-test. Statistical significance was set at *p* < 0.05.

## 3 Results

### 3.1 Sequencing data processing

The raw data and quality control results for all samples after being subjected to double-end sequencing on the Illumina NovaSeq platform are shown in Supplementary Table 1. Raw off-machine data yielded an average of 87,621 sequences per sample. After splicing and filtering low-quality and short-length sequences, chimeras were removed, and an average of 50,627 high-quality sequences with an average length of 418 bp were obtained. A total of 3,948,939 high-quality valid sequences were obtained for subsequent analysis.

### 3.2 OTUs clustering and species annotation

**Figure 1:**
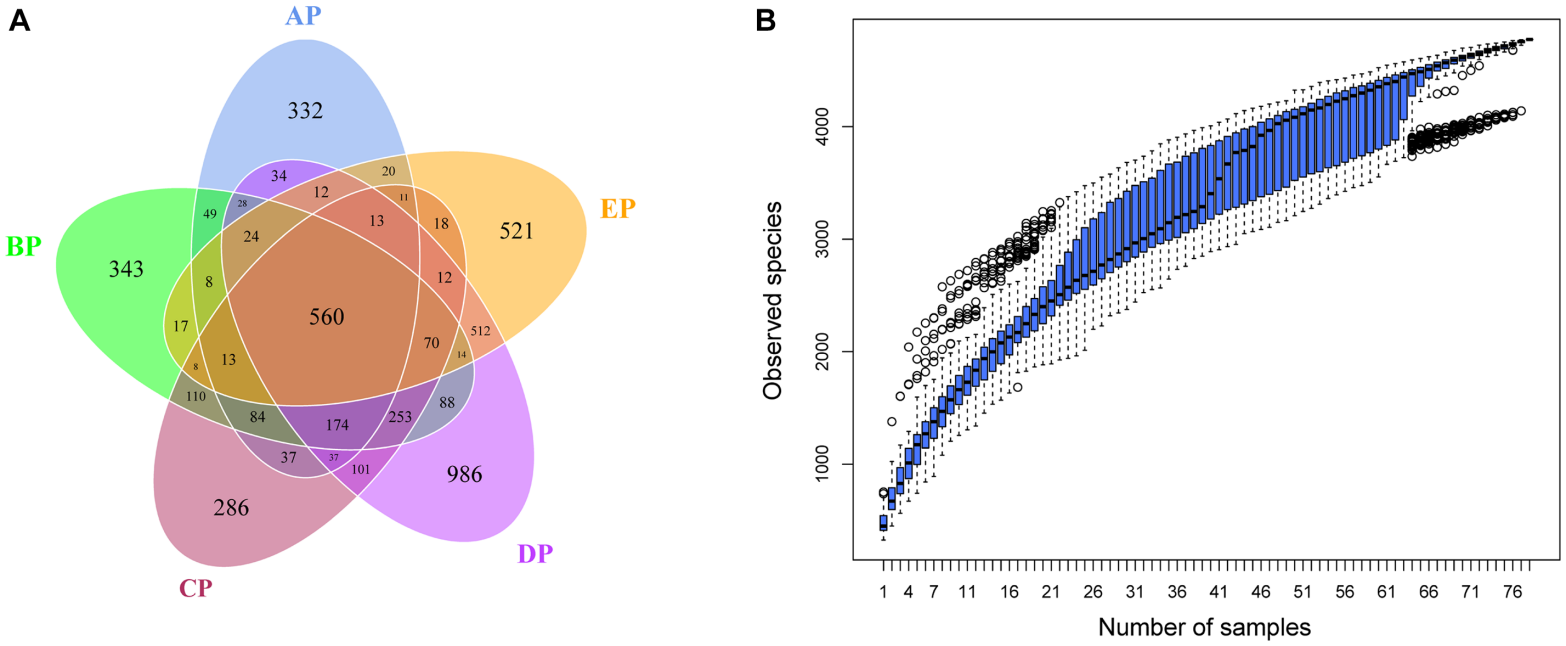
**(A)** Venn diagram of supragingival plaque samples. The numbers in the overlapping part of the circle represent the number of OTUs common to the group, and the numbers in the non-overlapping part represent the number of OTUs unique to the group. **(B)** Box plot of species accumulation in the supragingival plaque samples.

OTUs clustering analysis was performed on valid sequence data from supragingival plaque samples according to 97% concordance to analyze the composition and distribution of species in the supragingival plaque samples (Supplementary Figure 1). The clustering results showed that 4,941 OTUs were obtained, and 4,080 (82.57%) were annotated to the database. The OTUs at each level represented 2 bacterial boundaries, 29 phyla, 44 classes, 96 orders, 176 families, 379 genera, and 412 species. A Venn diagram showed that 1,436, 1843, 1787, 2,918, and 1833 OTUs were identified in the AP, BP, CP, DP, and EP groups, respectively, after 97% consistency treatment. Five groups of overlapping regions, i.e., 560 OTUs, were shared by the five groups (Figure 1A). Analyzing five groups of unique OTUs, we found 332, 343, 286, 986, and 521 unique OTUs in the AP, BP, CP, DP, and EP groups, respectively. The number of unique OTUs in the DP group was significantly higher than in the AP group. The results of the cumulative box plot of species indicated that the sample size in this experiment was sufficient to meet the analytical requirements for species richness (Figure 1B).

### 3.3 Alpha diversity analysis

**Figure 2:**
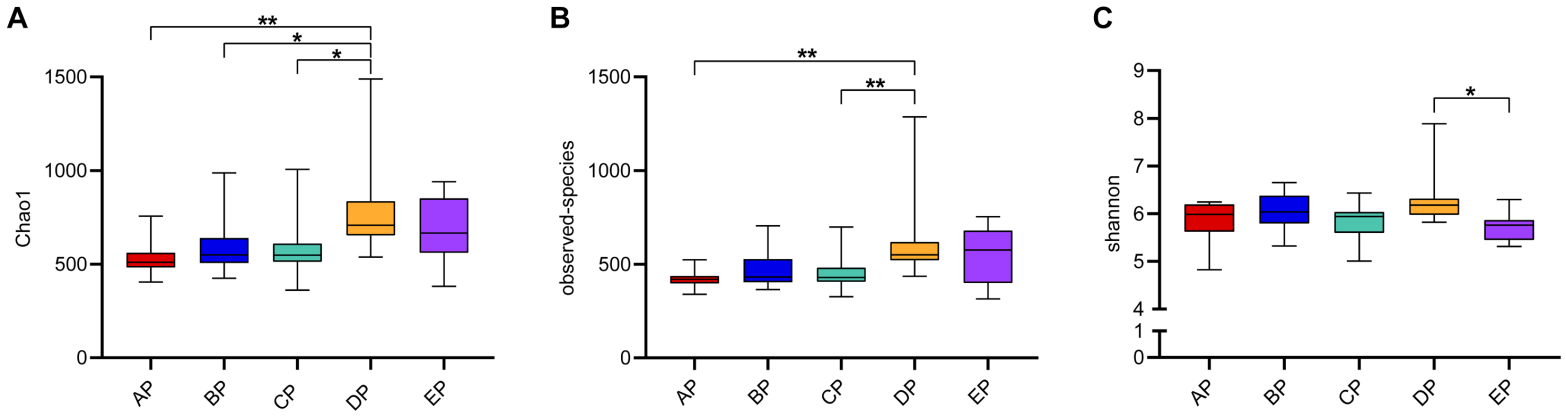
Box plot of Alpha diversity index of the supragingival plaque samples. The graphs show the Chao1 index **(A)**, observed species index **(B)**, and Shannon index **(C)**, respectively. The color boxes in the figure represent the middle 50% distribution interval of the group of data, and the upper and lower boundaries of the boxes are the upper and lower quartiles of the data, respectively; the middle line is the median. *p < 0.05, **p < 0.01.

Alpha diversity reflects species complexity within the sample community, and the results of each index are presented as box plots (Figure 2). The Chao1 index was used to estimate the number of species contained in the supragingival plaque samples, i.e., species richness. The results showed that the number of species in the samples showed an increasing trend with an increase in the-6° head-down bed rest experimental time compared to the AP group. The DP group had the largest number of species, which was significantly different from the AP, BP, and CP groups (*p* < 0.05). The number of species in the EP group did not differ significantly from those in the other four groups. The observed species index also showed that, at the same sequencing depth, the highest number of species was found in the DP group, which was significantly different from that in the AP group (*p* < 0.05). The Shannon index results showed no statistical difference in species diversity during HDBR compared with the AP group. Although species diversity in the EP group decreased significantly compared with the DP group (*p* < 0.05), it was not statistically different from that in the AP group. The above results showed that alpha diversity differed among the samples, with the DP group having the highest species richness.

### 3.4 Beta diversity analysis

**Figure 3:**
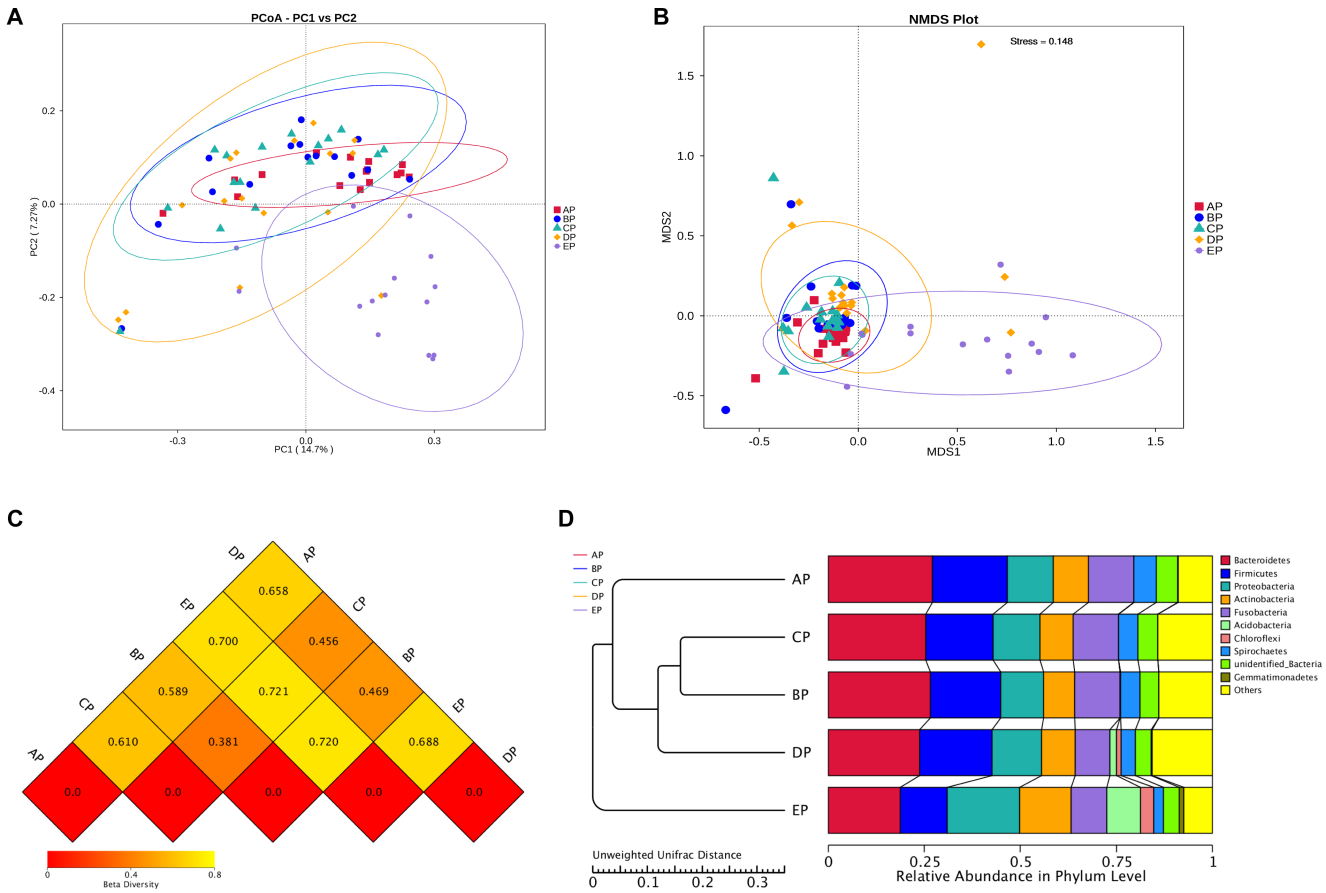
Beta diversity index plots of the supragingival plaque samples. **(A)** Two-dimensional principal coordinate analysis based on the Unweighted Unifrac distance. Horizontal coordinate, main component PC1; vertical coordinate, main component PC2. **(B)** Non-metric multidimensional scaling (NMDS) analysis based on the Bray-Curtis distance: Stress is less than 0.2, indicating that NMDS can accurately reflect the degree of dissimilarity among samples. **(C)** Heat map of the Beta diversity index. The numbers are the dissimilarity coefficients of the two groups. The reference values at the bottom right are the Beta diversity index values and the color shades correspondingly. **(D)** Unweighted pair-group method with arithmetic mean clustering tree.

Beta diversity analysis was performed to determine whether microgravity altered the microbial community structure. PCoA and NMDS analyses showed that the EP group was distinct from the other four groups in different directions, and the community structure was quite different (Figures 3A,B). The beta diversity index heatmap and UPGMA cluster analysis showed that the community structures of the AP and EP groups were quite different from those of the other four groups; however, the structures of the three groups during HDBR were similar (Figures 3C,D). To further verify the correctness of the above results, we also performed tests for differences between the groups (Table 1). These results showed that the community structures of the AP and EP groups were significantly different from those of the other four groups of samples under a simulated microgravity environment (*p* < 0.05).

**Table 1:** Dissimilarity analysis of supragingival plaque samples between groups.

Group	Anosim		Adonis		Amova	
	*R*	*p*	*R*2	*p*	Fs	*p*
AP-BP	0.1012	0.016	0.06707	0.017	1.33637	0.094
AP-CP	0.1364	0.003	0.07504	0.003	1.80369	0.011
AP-DP	0.1304	0.005	0.07517	0.004	2.58057	<0.001
AP-EP	0.1746	0.001	0.08985	0.006	3.68565	<0.001
BP-CP	0.02656	0.219	0.05628	0.07	0.673817	0.905
BP-DP	0.00344	0.378	0.04035	0.204	1.04837	0.34
BP-EP	0.1966	0.001	0.09544	0.002	4.06971	<0.001
CP-DP	0.08408	0.036	0.06574	0.012	1.14806	0.253
CP-EP	0.2694	0.002	0.11565	0.003	4.83831	<0.001
DP-EP	0.1492	0.001	0.07624	0.004	4.29182	<0.001

Anosim analysis and Adonis analysis based on Bray-Curtis distance; Amova analysis based on Unifrac distance. In Anosim analysis, *R* > 0 indicates significant differences between groups, and *R* < 0 indicates that intra-group differences are greater than inter-group differences. In Adonis analysis, a larger value of R2 indicates a higher degree of explanation of differences by group. In Amova analysis, Fs is the F-test value. The reliability of the above statistical analyses were expressed as *p* values, and *p* < 0.05 indicated that the statistical differences were significant.

### 3.5 Species distribution

**Figure 4:**
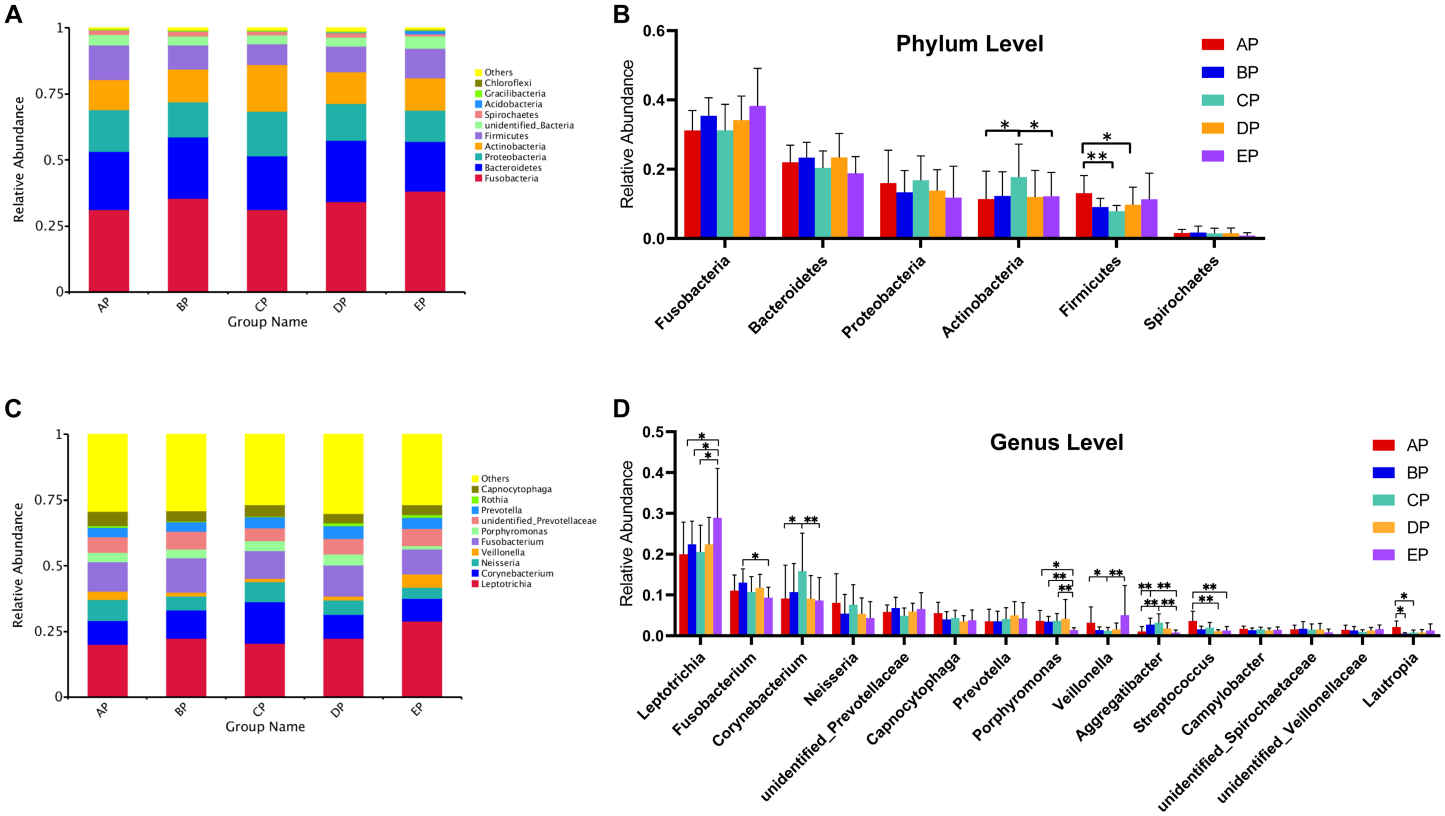
**(A)** Columnar accumulation plot for the top 10 phyla in relative abundance. **(B)** Differential analysis of the dominant phyla. **(C)** Columnar accumulation plot for the top 10 genera in relative abundance. **(D)** Differential analysis of the dominant genera. Others represent the sum of relative abundance of all species other than the first 10 species in the graph. *p < 0.05; **p < 0.01.

According to the species annotation results, 28 phyla were identified in the five groups of supragingival plaques at the phylum level. Columnar accumulation plots of the relative abundance of species drawn from the top 10 phyla with the maximum abundance were selected (Figure 4A). Among them, there were six dominant phyla (mean relative abundance >1%): Fusobacteria, Bacteroidetes, Proteobacteria, Actinobacteria, Firmicutes, and Spirochaetes according to their relative abundances from high to low, and the total abundance of the dominant phyla was 94.74%. Statistical analysis of differences in the relative abundance of dominant phyla revealed that compared to the AP group, Actinobacteria were significantly increased in the CP group (*p* < 0.05), whereas Firmicutes were significantly decreased in the CP (*p* < 0.01) and DP groups (*p* < 0.05); Actinobacteria were significantly decreased in the EP group compared with the CP group (*p* < 0.05) (Figure 4B).

In total, 379 genera were identified. Columnar accumulation plots of the relative abundance of the species were drawn for the top 10 most abundant genera (Figure 4C). There were 15 dominant genera with a total abundance of 80.10%. Statistical analysis of differences in the relative abundance of dominant genera revealed that compared with the AP group, *Aggregatibacter* were significantly increased (*p* < 0.01) and *Lautropia* were significantly decreased (*p* < 0.05) in the BP group; *Corynebacterium* (*p* < 0.05) and *Aggregatibacter* (*p* < 0.01) were significantly increased, *Veillonella* and *Lautropia* were significantly decreased (*p* < 0.05) in the CP group; *Streptococcus* were significantly decreased (*p* < 0.01) in the DP group; and *Leptotrichia* were significantly increased (*p* < 0.05), *Porphyromonas* (*p* < 0.05), and *Streptococcus* (*p* < 0.01) were significantly decreased in the EP group. Compared with the BP group, the EP group showed a significant increase in *Leptotrichia* (*p* < 0.05) and a significant decrease in *Fusobacterium* (*p* < 0.05) and *Porphyromonas* (*p* < 0.01). Compared to the CP group, the EP group showed a significant increase in *Leptotrichia* (*p* < 0.05) and *Veillonella* (*p* < 0.01) and a significant decrease in *Corynebacterium*, *Porphyromonas*, and *Aggregatibacter* (*p* < 0.01) (Figure 4D).

### 3.6 Genus-level species phylogenetic tree

**Figure 5:**
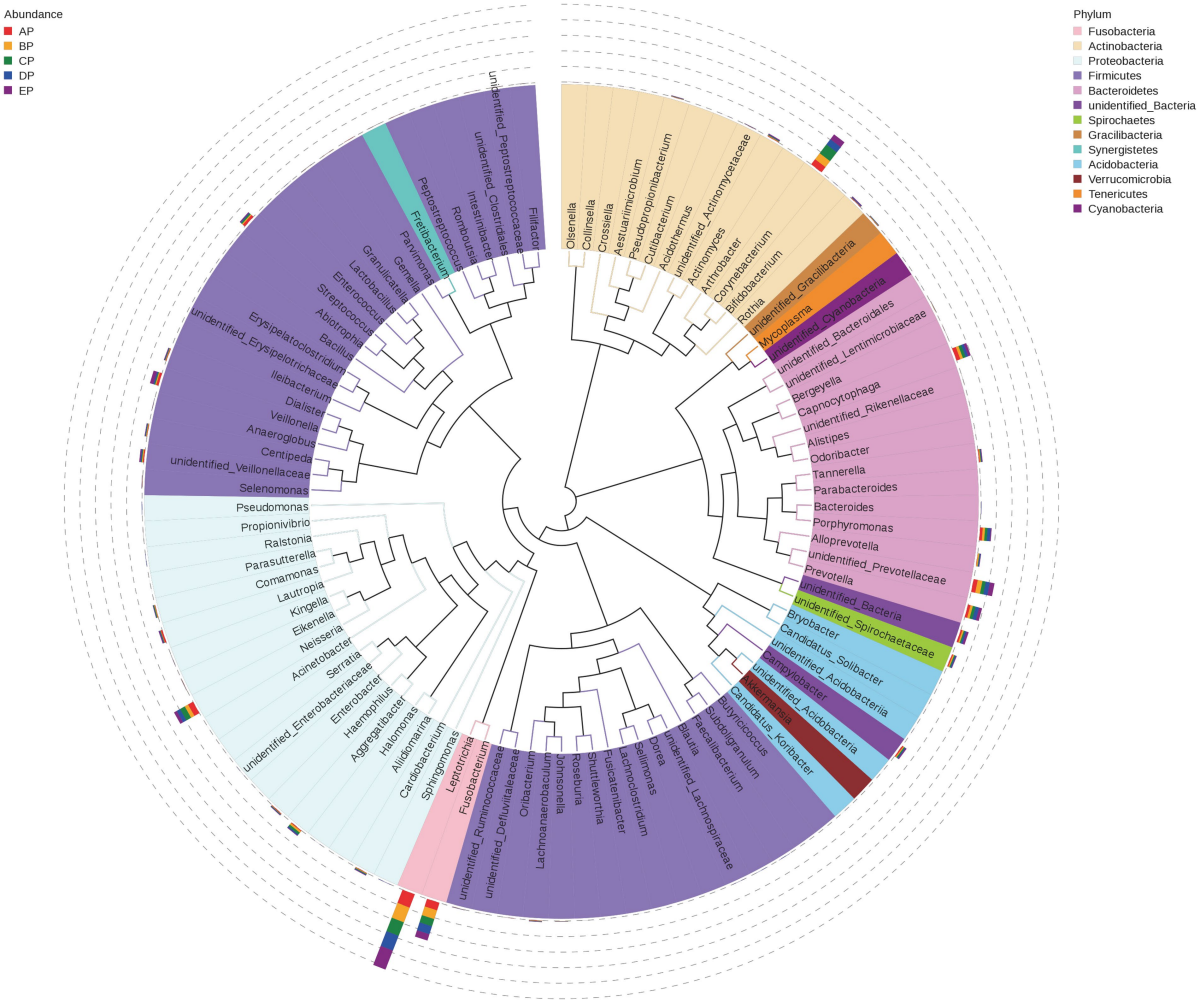
Phylogenetic tree of species at the genus level for supragingival plaque samples. The outer bar shows information on the abundance distribution of the genera in each of the five groups of samples, as shown in the left panel note. The colors of the branches and sectors indicate their corresponding phylum, as shown in the right panel note. The innermost tree structure refers to the OTU phylogenetic tree: each branch represents a species, and the length of the branch is the evolutionary distance between two species, i.e., the degree of species difference.

Representative sequences of the top 100 dominant and abundant genera in the supragingival plaque microorganisms were analyzed, and the microbial communities in all samples were mainly composed of Firmicutes, Proteobacteria, Actinobacteria, Bacteroidetes, and Fusobacteria. *Veillonella* in Firmicutes; *Neisseria* in Proteobacteria; *Corynebacterium* in Actinobacteria; *unidentified Prevotellaceae*, *Capnocytophaga*, *Prevotella,* and *Porphyromonas* in Bacteroidetes; and *Leptotrichia* and *Fusobacterium* in Fusobacteria contributed more to the species composition (Figure 5).

### 3.7 Network analysis

Network relationship diagrams demonstrate the interaction relationships among microbial genera in different states. The interactions among microorganisms in the five groups of supragingival plaque samples were obtained by calculating the Spearman correlation coefficient for all samples and removing the filtering conditions, which were visualized using a network diagram (Supplementary Figure 2). The connections among the nodes represent a significant correlation among the genera. The AP group contained 64 nodes and 180 connectors, the BP group contained 82 nodes and 264 connectors, the CP group contained 70 nodes and 182 connectors, the DP group contained 98 nodes and 577 connectors, and the EP group contained 78 nodes and 266 connectors. It can be seen that the correlation of genera became progressively more complex in all samples during HDBR and did not return to pre-HDBR levels in the EP group. Furthermore, we analyzed the interactive relationships among the 15 dominant genera (Supplementary Figure 3). There were 17 pairs of interrelationships in the AP group, 13 in the BP group, 16 in the CP group, 5 in the DP group, and 16 in the EP group. With these data, we found that the interrelationship between the dominant genera became simpler during HDBR. Simultaneously, the positive and negative correlations between the dominant genera changed dynamically, and the specific correlation coefficient values are listed in Table 2. No correlation was observed between Porphyromonas, Prevotella, and an unidentified Spirochaet*aceae* in the AP group, whereas a positive correlation emerged in the DP group. *Neisseria* showed a positive correlation with *Capnocytophaga* in both the AP and EP groups; however, this was not observed during the experimental period. *Fusobacterium* and *unidentified Prevotellaceae* showed negative correlations in the AP group and positive correlations in the CP group. These results indicated that the correlations among the microbial communities changed under simulated microgravity conditions.

**Table 2:** Correlations among the dominant genera in five groups.

Dominant genera		*R*				
		AP	BP	CP	DP	EP
*Leptotrichia*	*Fusobacterium*	−0.67	–	–	–	–
	*Neisseria*	−0.66	–	−0.67	–	–
	*unidentified_Prevotellaceae*	0.71	–	–	–	–
	*unidentified_Bacteria*	0.79	–	–	–	–
	*unidentified_Veillonellaceae*	0.73	–	–	–	0.66
	*Lautropia*	−0.66	–	–	–	–
*Fusobacterium*	*Corynebacterium*	–	–	−0.69	–	−0.60
	*unidentified_Prevotellaceae*	−0.63	–	0.64	–	–
	*Porphyromonas*	–				–	–	–	0.66
	*unidentified_Veillonellaceae*	−0.61	–				–	–	–
*Corynebacterium*	*Capnocytophaga*	–	0.75	–			–	–
	*Prevotella*	–	−0.70	−0.66	–	−0.65
	*Porphyromonas*	–	–	−0.62	–	–
	*unidentified_Spirochaetaceae*	–	–	−0.64	–		–
*Neisseria*	*unidentified_Prevotellaceae*	−0.84	−0.78	−0.66			–	–
	*Capnocytophaga*	0.63	–			–	–	0.85
	*Prevotella*	–	–	−0.71	–	−0.76
	*Streptococcus*	–	0.69	–	–	0.62
	*Campylobacter*		–	−0.60	–			–	–
	*unidentified_Spirochaetaceae*		–	–			–	–	−0.68
	*unidentified_Veillonellaceae*	−0.86	–				–	–	–
	*Lautropia*	0.72					–	–	–	–
*unidentified_Prevotellaceae*	*Capnocytophaga*	–		–	−0.66	–		–
	*Prevotella*			–	–			0.81	–	–
	*unidentified_Bacteria*	0.68	–				–	–	–
	*Streptococcus*	–	−0.83	–			–	–
	*Campylobacter*		–				0.80	–	–	–
	*unidentified_Spirochaetaceae*		–	–	0.65	–	–
	*unidentified_Veillonellaceae*	0.71	0.66	0.61	0.61	–	
	*Lautropia*	−0.65	–				–	–	–
*Capnocytophaga*	*Prevotella*	–		–	−0.61	–	−0.75
	*Aggregatibacter*			–	–		0.62	–	–
	*unidentified_Spirochaetaceae*			–	–		−0.69	–	−0.60
	*unidentified_Veillonellaceae*	−0.71	–				–	–	–
	*Lautropia*	–				–	–	–	0.75
*Prevotella*	*Porphyromonas*	–			–	–	0.75	–
	*unidentified_Spirochaetaceae*	0.82	–	0.82	0.87	0.74
	*Lautropia*	–			–	–	–	−0.65
*Porphyromonas*	*unidentified_Spirochaetaceae*				–	–	–	0.76	–
*Veillonella*	*Campylobacter*	–	0.67	–		–	–
*unidentified_Bacteria*	*Aggregatibacter*	–	−0.84			–	–	−0.64
	*unidentified_Veillonellaceae*	0.62	0.61	–			–	–
*Streptococcus*	*Campylobacter*	–	−0.73	–	−0.65	–
	*unidentified_Spirochaetaceae*		–	−0.67	–			–	–
	*unidentified_Veillonellaceae*		–	−0.77				–	–	–
	*Lautropia*	0.78	–				–	–	–
*Campylobacter*	*unidentified_Veillonellaceae*	–	0.68	–	–	0.61
*unidentified_Veillonellaceae*	*Lautropia*	−0.69	–				–	–	–

Spearman’s correlation coefficient is expressed by *R* value, and the table only shows data with correlation coefficient |R| > 0.6 and *p* < 0.05 between two dominant genera.

### 3.8 PICRUSt functional prediction

**Figure 6:**
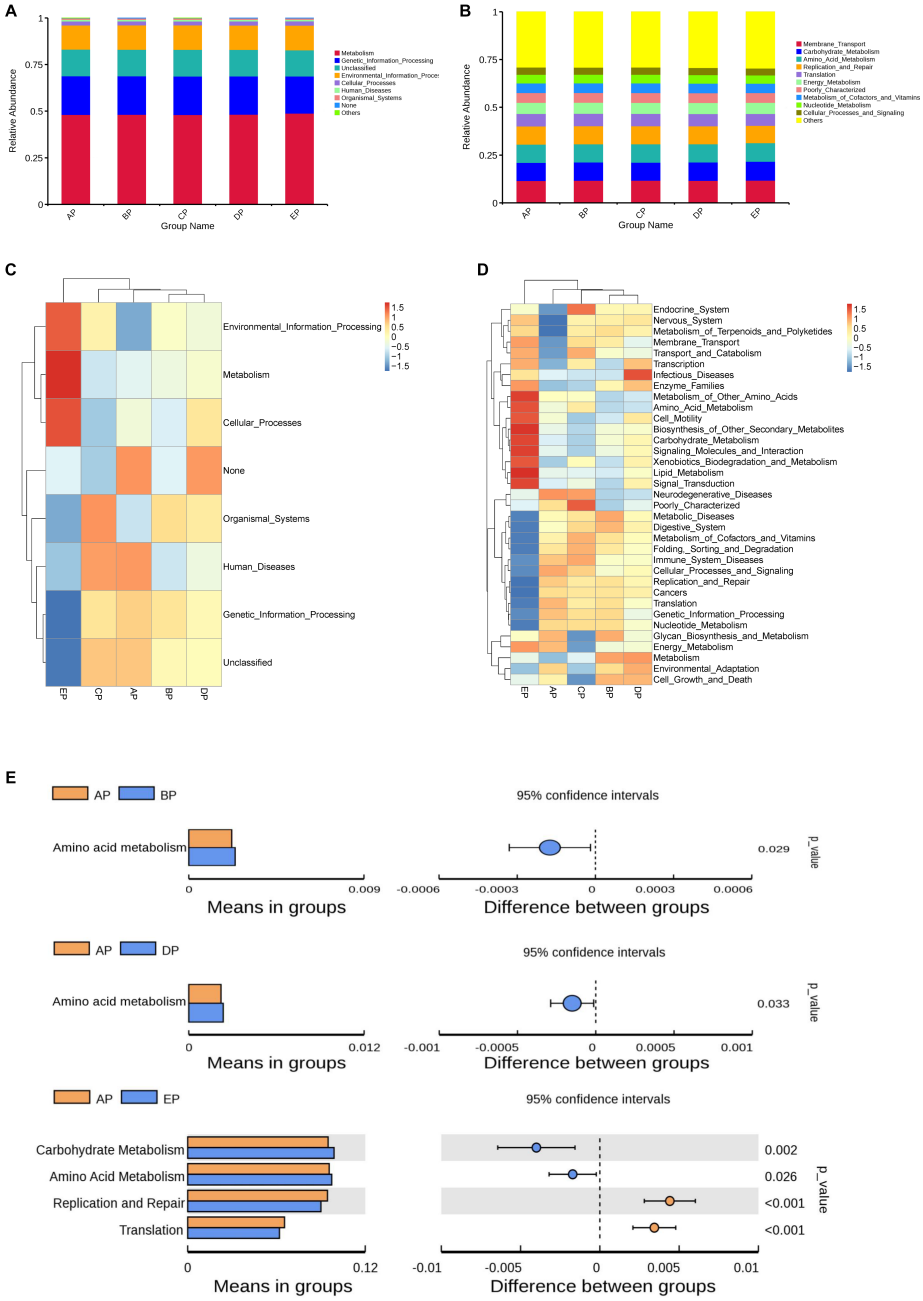
Functional genes of the samples were analyzed using the KEGG database. **(A,B)** Histograms of functional relative abundance at the first and second annotation levels. Others indicate the sum of relative abundance of all pathways other than metabolic pathways in the graph. **(C,D)** Heatmap of the top 35 functional genes in terms of relative abundance; the reference values on the top right are the correspondence between relative abundance and color shades. **(E)** Analysis of differences in functional genes based on the *t*-test. Each bar in the figure indicates the mean value of functional genes with significant differences in abundance between groups in each group, respectively. The right side shows the confidence level of the difference between groups, the leftmost point of each circle in the figure indicates the lower limit of the 95% confidence interval of the mean difference, and the rightmost point of the circle indicates the upper limit of the 95% confidence interval of the mean difference. The center of the circle represents the difference of means. The group represented by the color of the circle is the group with the highest mean. The rightmost point of the displayed results indicates the value of *p* of the intergroup significance test corresponding to the difference species.

The Kyoto Encyclopedia of Genes and Genomes (KEGG) database was used to analyze functional genes. Based on the annotation results of the database, the top 10 functional information of each grouping in terms of maximum abundance at each annotation level was selected to generate a functional relative abundance histogram (Figures 6A,B). The main functional gene categories were the same for all groups. According to the functional annotation and abundance information of the samples in the database, the top 35 abundant functions and their abundance information in each sample were selected to draw a heatmap, and clustering was performed based on the functional difference level (Figures 6C,D). Differences between the groups were analyzed based on the annotation results (Figure 6E).

The results of the first annotation hierarchy showed that the KEGG pathways for supragingival plaque microbes were mainly focused on metabolic and genetic information processing. Compared to the AP group, no significant difference was observed in gene function during HDBR; however, the metabolic function was more abundant, and the genetic information processing function decreased significantly in the EP group.

The second annotation hierarchy results showed that the top 10 major functional gene categories in relative abundance were the same for the five groups of samples, including membrane transport, carbohydrate metabolism, amino acid metabolism, replication and repair, and translation. Among them, the most abundant membrane transport function did not differ among the five groups; however, compared with the AP group, carbohydrate metabolism function was richer in the EP group, and amino acid metabolism function was richer in the BP, DP, and EP groups, although replication and repair function and translation function decreased in the EP group.

## 4 Discussion

Dysbiosis of the oral microbiota can lead to the development of several oral diseases. Therefore, exploring how changes in environmental factors can cause changes in the oral microbiota is crucial.

When clustering OTUs, we found a significant increase in the number of unique OTUs in the supragingival plaque microbial community on day 15 of HDBR compared to pre-HDBR. Meanwhile, Chao1 and the observed species indices showed a significant increase in species richness on day 15 of HDBR. This suggests that the number of species in the microbial community of the supragingival plaque increases under simulated microgravity. Ilyin (2005) collected oral microorganisms from astronauts at the Salyut and Mir orbital stations and found that the number of opportunistic pathogens increased during spaceflight, particularly the massive invasion of *Actinomyces_naeslundii*, *Prevotella_melaninogenica*, and *Fusobacterium_nucleatum* as well as bacterial species capable of maintaining the persistence of inflammation, but declined to preflight levels on day 14 after the return. Urbaniak et al. (2020) used 16S rRNA gene sequencing to analyze salivary microbes in 10 astronauts during missions of 2–9 months and observed that species richness increased significantly during space flight, but declined to preflight levels 6 months after returning to Earth. These studies are consistent with our experimental results, showing that the species richness of microorganisms increases significantly in microgravity environments. However, in this study, species richness did not return to pre-HDBR levels on day 6 of recovery, which may be because we did not observe species richness sufficiently long after HDBR. The Shannon index reflects the diversity of species, i.e., the combination of richness and evenness. The results showed no differential changes in microbial diversity during HDBR compared to pre-HDBR. Species diversity showed a significant decrease on day 6 of recovery compared to that during HDBR; however, considering no differences compared to pre-HDBR, we believe that species diversity returned to pre-HDBR levels on day 6 of recovery. Beta diversity analysis revealed significant differences in the structure of the supragingival plaque microbial community before and during HDBR and in the recovery state. Our group previously used 16S rRNA gene sequencing to examine saliva samples from volunteers under simulated microgravity conditions and found that the structure of the microbial community during HDBR differed from that before HDBR (Sun et al., 2022). However, Sun et al. (2022) did not observe differences in the abundance of salivary microbial communities during HDBR compared to before HDBR. This suggests that the microbial community changes in the free state saliva and adherent state supragingival plaque in the oral cavity are different in microgravity environments. This further demonstrates the necessity of exploring the changes in different oral microniches under simulated microgravity environments.

Species distribution was analyzed among the different groups, and we observed that the relative abundance of supragingival plaque microorganisms differed at different time points. The relative abundances of *Corynebacterium* and *Aggregatibacter* increased significantly during HDBR compared to pre-HDBR, and both declined to pre-HDBR levels on day 6 of recovery. One study found that *Corynebacterium* plays a critical role in plaque formation and underlies plaque structure and community interactions (Schoilew et al., 2019). Metagenomic sequence analysis showed that *Corynebacterium* is a key taxon of supragingival plaques, constituting a hedgehog-like structure and providing a structural backbone for the aggregation of other bacteria (e.g., *Streptococcus* and *Porphyromonas*), thereby forming a polymicrobial complex (Mark Welch et al., 2016; Fakhruddin et al., 2019). Chen et al. (2021) collected oropharyngeal samples from four healthy volunteers in a 180-day ground-simulated space-enclosed environment and found *Corynebacterium* as the dominant genus, whose relative abundance increased significantly throughout the experiment. In this study, a significant increase in the relative abundance of *Corynebacterium* during HDBR may have facilitated the aggregation of more pathogenic microorganisms. *Aggregatibacter* is gram-negative bacilli that is integral to healthy oral microbial communities; however, in conditions such as trauma or mucosal damage, it invades healthy tissues, causing infection. Members of this genus identified in our analysis were *Aggregatibacter segnis* and *Aggregatibacter actinomycetemcomitans*. *A. actinomycetemcomitans* is a well-known human periodontal pathogen (Fine et al., 2019). *A. actinomycetemcomitans* can produce various virulence factors, such as leukotoxins and cell expansion lethal toxins, destroy the local immunity of the host, and help periodontal pathogens evade host immunity, leading to the occurrence and development of periodontal disease (Shenker et al., 1999; Kachlany, 2010). Simultaneously, *A. segnis* has been suggested to have adhesiveness to the cellular structure and coexists with other bacteria, causing supragingival calculus formation (Baris et al., 2017).

In contrast, the relative abundances of *Veillonella, Lautropia,* and *Streptococcus* decreased significantly during HDBR compared to pre-HDBR. On day 6 of recovery, the relative abundances of *Veillonella* and *Lautropia* increased to pre-HDBR levels, whereas the relative abundance of *Streptococcus* remained different from that of pre-HDBR. Evidence exists that *Veillonella* lacks glycolytic capacity, relies solely on lactic acid and other organic acids as energy sources, produces lower pKa acids, and may increase the pH, suggesting that *Veillonella* may have beneficial effects on the oral environment (Gross et al., 2010). Quantitative data suggest a reduction in the frequency index of isolation of *Veillonella* gingival crevicular fluid of astronauts on day 1 after space flight (Volozhin et al., 2001). Similarly, *Lautropia* is a normal oral cavity colonizer and is the dominant genus in healthy oral environments (Ikeda et al., 2020; Fragkioudakis et al., 2021). Individuals with periodontal disease have been observed to exhibit a decrease in the content of *Lautropia* (Shaddox et al., 2012). *Streptococcus* species consists of numerous opportunistic pathogens associated with dental caries. Cheng et al. (2014) found that simulating microgravity conditions can change the structure of *Streptococcus mutans* biofilms and the distribution of extracellular polysaccharides and improve the acid resistance of *S. mutans*. Brown et al. (1974) detected a significant increase in *S. mutans* counts in the dental plaque of three astronauts during a 56-day simulated space laboratory mission. Because of limited experimental conditions, they only examined quantitative changes in species.

Furthermore, a significant increase was observed in the relative abundance of *Leptotrichia* and a significant decrease in the relative abundance of *Porphyromonas* on day 6 of recovery compared to pre-HDBR and during HDBR. *Leptotrichia* is highly glycolytic and ferments a variety of monosaccharides and disaccharides into lactic acid (Birkeland and Hofstad, 1985; Thompson and Pikis, 2012). This implies that *Leptotrichia* may be involved in the development of dental caries and may have cariogenic potential. As an opportunistic pathogen, *Leptotrichia* is associated with infections, particularly in immunocompromised hosts (Eribe and Olsen, 2008; Eribe and Olsen, 2017). Moreover, Brown et al. (1976) found that 4 days after the end of a 59-day space laboratory mission, the number of *Leptotrichia* in the plaque of major aircrew increased significantly compared with that before the flight, which is consistent with our findings. *Porphyromonas* are genetically a highly heterogeneous group, and some species in this group, such as *P. pasteri* and *P. catoniae,* can be used as potential oral health markers (Crielaard et al., 2011; Yasunaga et al., 2017). However, other species, such as *P. gingivalis* and *P. endodontalis,* are closely related to the occurrence and development of oral diseases (Hajishengallis et al., 2012). The primary colonizing habitat of *P. gingivalis* in a normal oral environment is the sulcus (Reinhardt et al., 1989). However, the flow of crevicular fluid in microgravity environments may lead to the translocation of microorganisms. Shi et al. (2016) found through a rotating cell culture system that simulated microgravity promotes the growth of *P. gingivalis* and alters its gene expression.

Network analysis showed that the interaction relationships between the 15 dominant genera in the supragingival plaque microbial community became simpler during HDBR than during pre-HDBR; however, new correlations emerged. We observed no correlation between *Porphyromonas* and *Prevotella* pre-HDBR or between *Porphyromonas* and *unidentified Spirochaetaceae*; however, a positive correlation was observed on day 15 of HDBR. The co-aggregation between *gingivalis* and *Prevotella_oris* has been proposed as a factor that promotes the advancement of periodontitis (Sato and Nakazawa, 2014). Simultaneously, *P. intermedia* exerted a synergistic effect with *P. gingivalis W83* and exacerbated the virulence of *P. gingivalis* by regulating the expression of its virulence factors (Zhang et al., 2019). Moreover, a synergistic growth relationship was observed between *P. gingivalis* and *T. denticola* (Huang et al., 2011). *P. gingivalis* produces isobutyric acid, which promotes the growth of *T. denticola* (Grenier, 1992). *T. denticola* produces succinic acid found in the phospholipids and lipids on the cell envelope of *P. gingivalis* (Lev, 1979). Therefore, we believe that alterations in the interactions between microbes may disrupt the balance of the oral microbial community structure and increase the risk of opportunistic infections. The impact of the interactions between these microbes should not be underestimated.

We also explored the alterations in microbial gene function in supragingival plaques under simulated microgravity. We found that amino acid metabolism was more abundant in the supragingival plaque microbes during HDBR, and this alteration persisted until day 6 of recovery. Current studies suggest that a complex regulatory network exists for amino acid metabolism and that acidic, basic, aromatic, and sulfur-containing amino acid metabolites produced by oral microorganisms through amino acid metabolism may negatively affect the host and exert pro-inflammatory and cytotoxic effects (Barbour et al., 2022). Moreover, environmental factors can alter the metabolic properties of certain bacteria, such as *Porphyromonas gingivalis*, *Prevotella intermedia*, and *Veillonella*, allowing them to exert different pathogenicity (Li et al., 2022). Therefore, we believe that the metabolic balance between microorganisms and their hosts plays an important role in maintaining a stable state of the body. The gene function of supragingival plaque microorganisms changed under the influence of a simulated microgravity environment, and this change persisted during the recovery period, which may lead to disturbances in oral microecology.

Overall, this study combined high-throughput sequencing technology with simulated microgravity experiments to comprehensively analyze the gene sequences of supragingival plaque microorganisms. We chose multiple time points to investigate changes in the microbial community under simulated microgravity conditions. Moreover, we explored the functions of the microbial genome to provide new insights for future studies. In the future, we will combine microbiome changes with laboratory findings to conduct metabolomic studies of supragingival plaque microorganisms in a broader population, further establish disease metabolic regulatory networks, lay the foundation for establishing *in vivo* and *ex vivo* research targets for the effects of microgravity environments on oral supragingival plaque microorganisms, and deeply explore the relationship between oral dysbiosis and immune disorders and systemic changes in the microgravity environment, providing a theoretical basis for the prevention and treatment of oral aerospace diseases.

## 5 Conclusion

This experimental study showed that a simulated microgravity environment with a 15-day −6° head-down bed rest would have an effect on the supragingival plaque microbial community in young men. In this state, the richness of the plaque microbial communities was significantly altered, and the community composition differed between the groups. Simultaneously, the relative abundance and functional genes of the microorganisms changed. Some of these changes persisted until the end of the experiment. This suggests that the simulated microgravity environment may affect the oral ecosystem by changing the balance of the supragingival plaque microbial community, leading to infectious diseases. Astronauts need to focus on oral hygiene care during and after spaceflight, either through a balanced diet or supplementation with specific probiotics to maintain a balanced microbial community.

## Data availability statement

The original contributions presented in the study are publicly available. This data can be found here: [https://www.ncbi.nlm.nih.gov/, PRJNA1003027].

## Ethics statement

The studies involving humans were approved by the Ethics Review Committee of the Space Science and Technology Institute (Shenzhen). The studies were conducted in accordance with the local legislation and institutional requirements. The participants provided their written informed consent to participate in this study.

## Author contributions

DZ: Formal analysis, Validation, Writing – original draft, Writing – review & editing. PQ: Conceptualization, Investigation, Writing – review & editing. QZ: Conceptualization, Investigation. HS: Formal analysis. BX: Methodology, Resources. BW: Methodology, Resources. CT: Conceptualization, Project administration, Supervision, Writing – review & editing.

## Footnotes

^1^ http://www.arb-silva.de/
