# Supplementary material for: Effect of 15 days −6° head-down bed rest on microbial communities of supragingival plaque in young men
Source: Front Microbiol. 2024 Jan 24;15:1331023. doi: 10.3389/fmicb.2024.1331023 (PMC10849213; doi:10.3389/fmicb.2024.1331023)
Supplement: Supplementary file 2 [file Data_Sheet_1.PDF]

Supplementary Material

Supplementary Figures and Table

1 Supplementary Figures

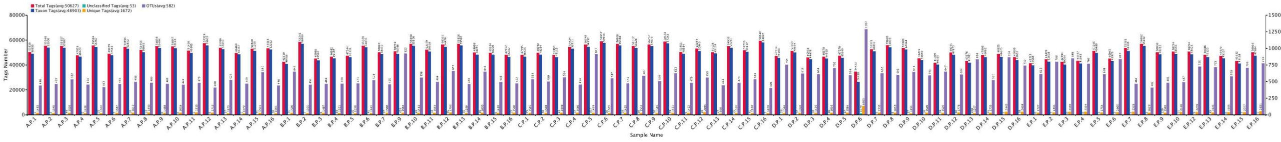

Supplementary Figure 1. Statistical results of the sequences and operational taxonomic units (OTUs) of supragingival plaque samples. The colors of the bar graph indicate the following: Total Tags, the number of valid sequences; Unclassified Tags, the number of sequences that can annotate OTUs but cannot clearly classify information; OTUs, the number of OTUs finally used for analysis; Taxon Tags, the number of sequences that can annotate OTUs and can clearly classify information; Unique Tags, the number of sequences that cannot be annotated as OTUs.

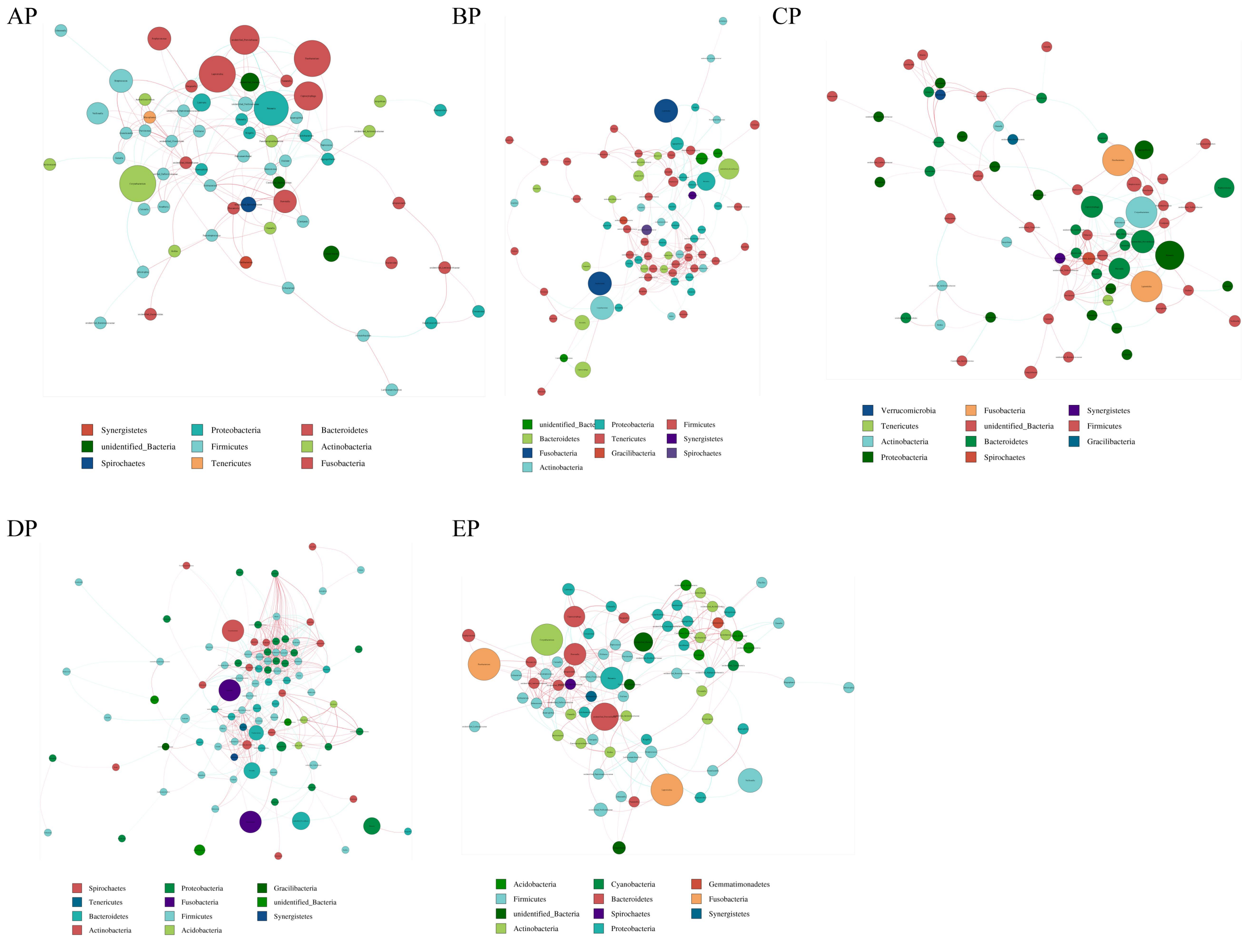

Supplementary Figure 2. Species relationship network diagrams between microorganisms in all five groups of samples. Each node represents a genus, the size of the nodes is determined by the mean relative abundance, the nodes at the same phylum level have the same color, the Spearman's correlation coefficient |R|>0.6 and P<0.05 between the nodes are connected by a line, the thickness of the line is positively correlated with the absolute value of the correlation coefficient of species interactions, the red line indicates positive correlation, the blue line indicates negative correlation.

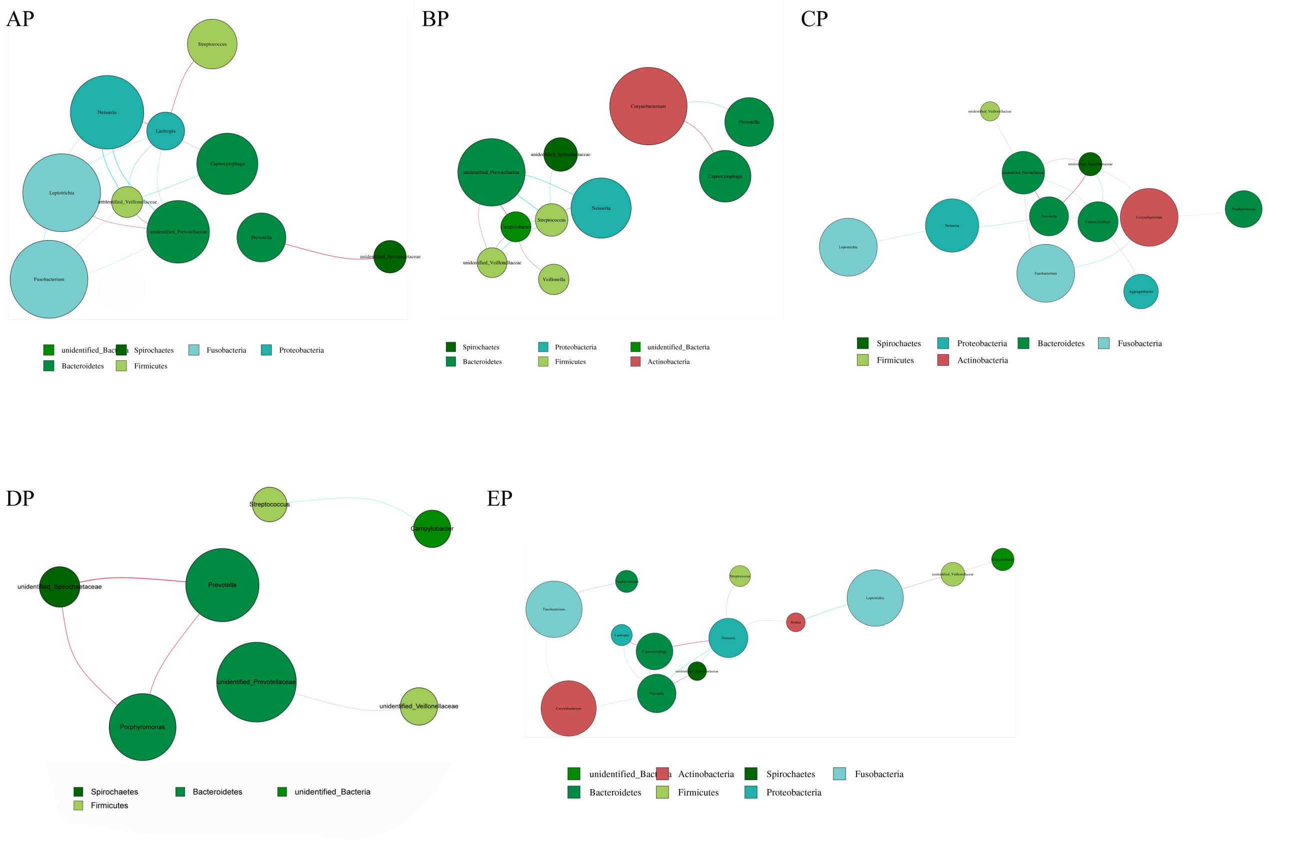

Supplementary Figure 3. Species relationship network diagram between dominant genera. Each node represents a genus, the size of the nodes is determined by the mean relative abundance, the nodes at the same phylum level have the same color, the Spearman's correlation coefficient $|R|>0.6$ and $P<0.05$ between the nodes are connected by a line, the thickness of the line is positively correlated with the absolute value of the correlation coefficient of species interactions, the red line indicates positive correlation, the blue line indicates negative correlation.

2 Supplementary Table

Supplementary Table. The first column indicates the names of all samples; the data of A.P.8 and E.P.11 are excluded due to quality control failures. Raw PE: the raw sequencing data; Raw Tags: the sequence of tags obtained by splicing; Clean Tags: the sequence of tags obtained after filtering low-quality and short-length Raw Tags; Effective Tags: the sequence of tags finally used for subsequent analysis after filtering chimeras; Base: the number of bases in the final effective data. AvgLen: the average length of effective tags; Q20 and Q30: the percentage of bases with base quality values greater than 20 (sequencing error rate less than 1%) and 30 (sequencing error rate less than 0.1%) in effective tags; GC (%): the content of GC bases in effective tags; Effective (%): the percentage of the number of effective tags to the number of Raw PEs.
