# Supplementary figures and images for: Effect of 15 days −6° head-down bed rest on microbial communities of supragingival plaque in young men
Source: Front Microbiol. 2024 Jan 24;15:1331023. doi: 10.3389/fmicb.2024.1331023 (PMC10849213; doi:10.3389/fmicb.2024.1331023)

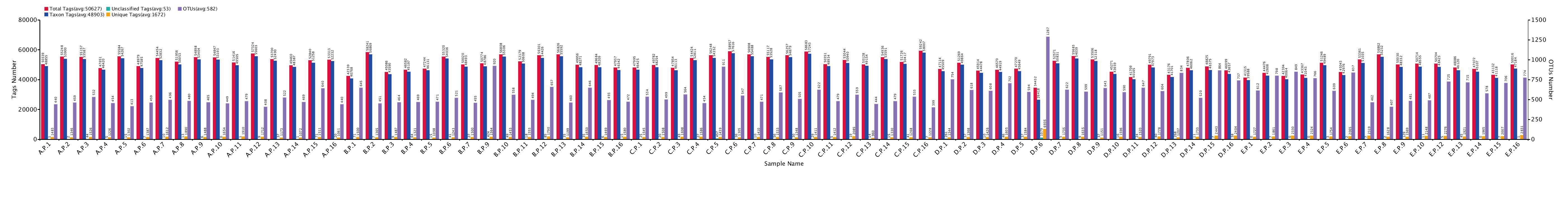

Supplement: Supplementary file 3 [file Image_1.TIF]
